# American Medical Association and Medical Education

**Published:** 1858-01

**Authors:** 


### "American Medical Association and Medical Education"

Under the head of Selections, will be found an article taken from the editorial department of the Memphis Medical Recorder, conducted by Prof. D. F. Wright, which contains so much more practical, good sense, upon the vexed question of medical education than we have met with in some other quarters, that we would commend it to the attention of those who have of late constituted themselves the special champions and peculiar defenders of the rights, powers and obligations of the American Medical Association.

Without entering again into the merits of the abstract question, (which we have already so fully discussed through our columns,) we would remark, that though connected with a Medical College, we are, perhaps, as little dependent upon the *business* of medical teaching, as some of those who boast more largely of their independence and freedom from selfish considerations in the present pseudo reform movement upon the subject of medical education.

We know that the origin and true character of the recent agitation of this question is fully understood by us, and unless we greatly mistake the indications, some of those who have "raised the wind will yet reap the whirlwind"—some of those who have been digging a pit for others, may yet find it their own resting place—some people who have been throwing stones may soon find the shattered fragments of their own houses toppling about their heads.

The warning which we have given, upon a former occasion, that "pride cometh before a fall," to which may be added, "set your own house in order, before going about to regulate those of others," we again commend to the attention of some people who are more under the influence of selfishness and a spirit of revenge, than the desire to elevate our noble profession.

We are most decided advocates of a more thorough system of medical instruction, as we are equally the advocates of sincerity and common sense, and are ready to meet this question, let it come in what shape it may.

---

☞ We find upon our table among a large number of addresses, monographs, and pamphlets of great variety which we have not been able to notice. "An Introductory Lecture upon Medical Education," delivered before the Memphis Medical College, on the 2d day of November, 1857, by A. P. Merrill, M. D., Professor of the Principles and Practice of Medicine, which, if we had space, it would afford us great pleasure to publish entire, as it contains many strong and striking views upon a number of points connected with this subject. We are much pleased to learn that the Memphis Medical College is now upon a stable basis, and is looking forward to a bright and successful future. We have intimated upon a former occasion, our opinions in reference to the advantages of Memphis, as a seat of medical learning, and hope to be able to chronicle the prosperity which the location and the merits of the institution should assuredly secure,

